# Selective participation of c-Jun with Fra-2/c-Fos promotes aggressive tumor phenotypes and poor prognosis in tongue cancer

**DOI:** 10.1038/srep16811

**Published:** 2015-11-19

**Authors:** Shilpi Gupta, Prabhat Kumar, Harsimrut Kaur, Nishi Sharma, Daman Saluja, Alok C. Bharti, Bhudev C. Das

**Affiliations:** 1 Department of Molecular Oncology, Dr. B.R. Ambedkar Centre for Biomedical Research (ACBR), University of Delhi, New Delhi-110007, India; 2 Amity Institute of Molecular Medicine & Stem Cell Research (AIMMSCR), Amity University Campus, Uttar Pradesh, Sector-125, Noida-201313, India; 3 Department of Otorhinolaryngology, Post Graduate Institute of Medical Education and Research, Dr. Ram Manohar Lohia (RML) Hospital, New Delhi-110010, India; 4 Division of Molecular Oncology, Institute of Cytology & Preventive Oncology (ICMR), Uttar Pradesh, Sector-39, Noida-201301, India

## Abstract

Tongue squamous cell carcinoma (TSCC) is most aggressive head and neck cancer often associated with HR-HPV infection. The role of AP-1 which is an essential regulator of HPV oncogene expression and tumorigenesis is not reported in tongue cancer. One hundred tongue tissue biopsies comprising precancer, cancer and adjacent controls including two tongue cancer cell lines were employed to study the role of HPV infection and AP-1 family proteins. An exclusive prevalence (28%) of HR-HPV type 16 was observed mainly in well differentiated tongue carcinomas (78.5%). A higher expression and DNA binding activity of AP-1 was observed in tongue tumors and cancer cell lines with c-Fos and Fra-2 as the major binding partners forming the functional AP-1 complex but c-Jun participated only in HPV negative and poorly differentiated carcinoma. Knocking down of Fra-2 responsible for aggressive tongue tumorigenesis led to significant reduction in c-Fos, c-Jun, MMP-9 and HPVE6/E7 expression but Fra-1 and p53 were upregulated. The binding and expression of c-Fos/Fra-2 increased as a function of severity of tongue lesions, yet selective participation of c-Jun appears to promote poor differentiation and aggressive tumorigenesis only in HPV negative cases while HPV infection leads to well differentiation and better prognosis preferably in nonsmokers.

Head and neck squamous cell carcinoma (HNSCC) is an extremely heterogeneous group of cancers arising from different subsites such as tongue, lips, larynx and other intra-oral locations^1^,^2^. This clinical heterogeneity in terms of the site of origin also correlates with specific risk-factors, symptoms, tendency to local and distant metastasis, sensitivity to chemo-radiotherapy and the disease prognosis^3^,^4^.

Among HNSCCs, tongue squamous cell carcinoma (TSCC) is one of the most common but highly aggressive cancer particularly in younger patients and is associated with a higher rate of metastasis with poor prognosis^5^,^6^,^7^. In India, the incidence of TSCC is second highest in the world^8^. While smoking, tobacco, betel nut chewing and alcoholism are primary risk-factors, studies have reported that infection of HPV particularly high-risk HPV (HR-HPV) type 16 may also act as an independent risk-factor in inducing a substantial proportion of tongue cancer^6^,^7^,^9^. It has been demonstrated that unlike cervical cancer, TSCCs including oral squamous cell carcinoma and other HNSCCs with HR-HPV infection show better prognosis^6^,^7^,^9^,^10^,^11^,^12^,^13^,^14^. This has been further shown to be due to selective participation of NF-κB p65 that induces well differentiation of tumors leading to better prognosis^11^.

It is well established that transcription factor activator protein-1 (AP-1) formed by homo or hetero-dimerization between Jun (c-Jun, Jun-B, Jun-D) and Fos (c-Fos, FosB, Fra-1, Fra-2) family proteins^15^ plays a central role in HPV oncogene expression and tumorigenesis^16^,^17^. A high DNA binding activity and differential overexpression of AP-1 family proteins have been reported in many cancers, suggesting a pivotal role of AP-1 in tumor progression and metastasis^14^,^18^,^19^,^20^,^21^,^22^. AP-1 activation is known to further upregulate various downstream target genes such as cyclin Dl, c-myc, Bcl-xl, MMP-9, EGFR, and specific miRNAs etc., that are acitively involved in progression, metastasis and aggressive phenotype of various tumors^14^,^19^,^20^,^21^.

Several studies have demonstrated differential expression and high DNA binding activity of specific members of AP-1, particularly c-Fos, c-Jun and JunB during development of variety of carcinomas including oral carcinoma^18^,^19^,^20^,^22^. In contrast, Fos-related antigen 1 (Fra-1) has been shown to overexpress only in normal but absent in cancer cases (except breast cancer) indicating its possible tumor suppressor activity in these tumors^20^,^22^,^23^ while the Fos-related antigen 2 (Fra-2) has been found to be often highly upregulated in many cancers which show aggressive tumor phenotype and metastasis^19^,^24^,^25^. Although, aberrant activation and differential expression pattern of AP-1 family proteins have been reported in many cancers including oral cancer^18^,^19^,^20^,^22^, to date, there is no study that defines the role of AP-1 and its family proteins during tongue carcinogenesis. Therefore, the present study has been carried out, to investigate the role of AP-1 and its family proteins in different stages of tongue cancer including its precancer lesions to understand the contribution of specific AP-1 family proteins in presence or absence of HPV infection and their crosstalk in aggressive tongue carcinogenesis. The results demonstrated a selective interaction of c-Jun with Fra-2/c-Fos in absence of HPV promotes aggressive and invasive tongue tumorigenesis and poor prognosis while HPV infection facilitates well differentiation and better prognosis.

## Results

A total of one hundred prospectively collected fresh tongue tissue biopsy specimens comprising precancer (n = 20), cancer (n = 50) and adjacent normal controls (n = 30) and two tongue cancer cell lines (UPCI:SCC090 and AW13516) were employed for the detection of HPV infection, HPV genotypes and analysis of expression and activation of AP-1 family proteins. The Clinico-epidemiological and demographical details along with status of HPV infection are presented in Table 1. The majority of tongue cancer patients (84%; 42/50) were smokers and males (n = 40) with a mean age of 40.48 ± 12.46 years.

### Exclusive prevalence of HR-HPV type 16 mainly in well differentiated tongue squamous cell carcinoma

The analysis of HPV infection and its genotype distribution in tongue cancer by HPV type-specific PCR and reverse line blot (RLB) assay demonstrated only 14 (28%) HPV^+ve^ cases out of 50 TSCCs studied and all of them were positive exclusively for HR-HPV type 16 and no other known HPV type could be detected (supplementary Fig. 1). All precancer cases and normal adjacent controls were also found to be negative for HPV (Table 1). Of twenty precancer cases, 55% (11/20) were leukoplakia/eyrthoplakia while 45% (9/20) were dysplasia. Out of all HPV^+ve^ TSCCs (n = 14), majority (57.2%; 8/14) of them were females. The majority (78.6%, p ≤ 0.01) of HPV infection was found in well differentiated tumors (WDSCCs) in comparison to MDSCCs and PDSCCs (see Table 1) and belonged to early stage tumors (stage I and II; 71.4%, p ≤ 0.01). Interestingly, a very low frequency of HPV infection was observed in TSCC patients who were smokers (19%; 8/42) when compared to nonsmokers (75%, 6/8; p ≤ 0.01), the majority of whom were HPV^+ve^ (Table 1).

### Higher DNA binding activity of AP-1 formed by heterodimerization of c-Fos/Fra-2 and selective participation of c-Jun

**Figure 1:**
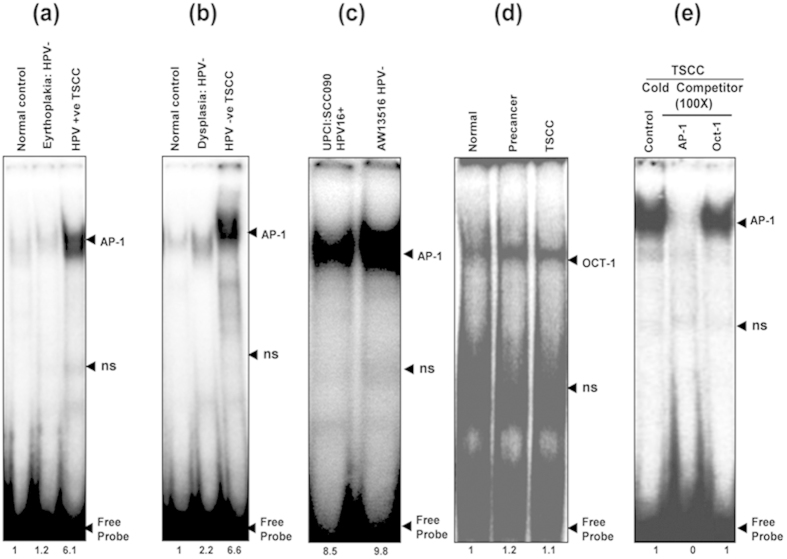
(**a**–**e**): Constitutive activation and higher DNA binding activity of AP-1 in HPV^+ve^ and HPV^−ve^ tongue tumor tissues and cell lines. EMSA showing DNA binding activity of AP-1 in nuclear extracts (10 μg) of different grades of tongue tissues (**a**,**b**) and cell lines (**c**) using γ^32^P- ATP-radiolabeled oligonucleotide harboring an AP-1 consensus sequence. Increasing AP-1 binding activity was observed as the severity of tongue lesions progressed from normal to precancer to invasive cancer in both HPV16^+ve^ (**a**) and HPV^−ve^ (**b**) cases. Both HPV16^+ve^ UPCI:SCC090 and HPV^−ve^ AW13516 (**c**) cell lines showed higher binding activity of AP-1. (**d**) EMSA with labeled Oct-1 probe showed uniform DNA binding in all grades of tongue tissues. Binding specificity was evidenced in a competition assay with nuclear extracts of TSCCs incubated with unlabelled 100 molar excess of specific competitor AP-1 probe and nonspecific competitor Oct-1 probe (**e**). Fold change in the band intensities of AP-1 are indicated in each lane. Ns: non-specific binding.

The DNA binding activity of AP-1 was analyzed by band shift assay in nuclear extracts derived from fresh tongue tissue biopsies of precancer (n = 20), cancer (n = 50) and normal adjacent control tissues (n = 30) as well as in two tongue cancer cell lines (UPCI:SCC090; HPV^+ve^ and AW13516; HPV^−ve^) using γ^32^P-labeled probe harboring AP-1 consensus sequence. As presented in Fig. 1a–c, a higher AP-1 DNA binding activity was detected in all tongue cancer cases and cell lines, whereas it was low or nil in normal adjacent controls but a moderate binding activity was observed in precancer lesions (Fig. 1a,b). EMSA showed a gradual increase in AP-1 binding activity with the increasing severity of the lesion but relatively a higher DNA binding was observed in HPV negative tumors (Fig. 1b). The specificity of binding activity was confirmed by performing cold competition assay with 100-fold molar excess of homologous AP-1 and a heterologous Oct-1 cold probe (Fig. 1d,e).

**Figure 2:**
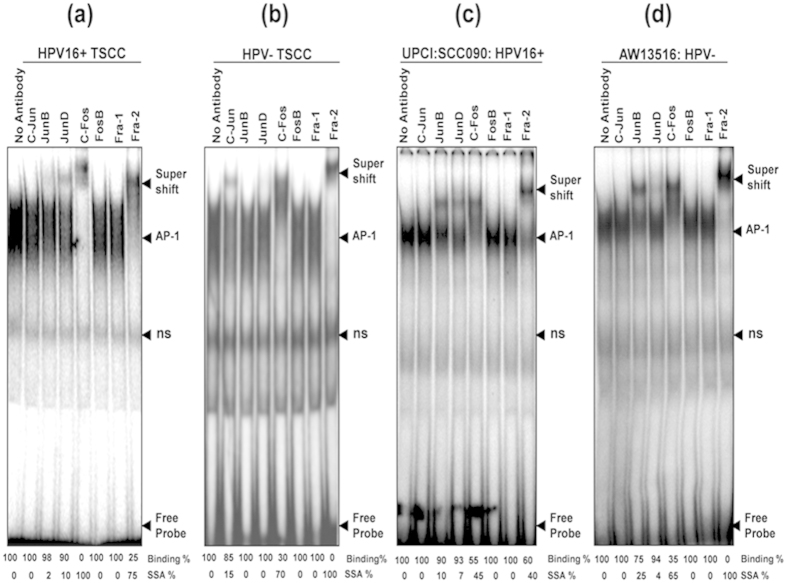
(**a**–**d**): Altered composition of functional AP-1 complex in HPV^+ve^ and HPV^−ve^ tongue tumor tissues and cell lines. Band supershift assay using specific antibodies (Abs) against all AP-1 family members (c-Jun, JunB, JunD, c-Fos, FosB, Fra-1, Fra-2). Panel a, b, c and d showing differential binding activity of AP-1 members in HPV^+ve^ (**a**) and HPV^−ve^ (**b**) TSCCs and tumor cell lines; UPCI:SCC090;HPV16^+ve^(**c**) and AW13516; HPV^−ve^(**d**). In all panels (**a–d**) significantly a higher (~90) binding of c-Fos and Fra-2 forms the functional AP-1 complex. HPV^+ve^ TSCC (panel **a**) shows a minor participation of JunD and JunB while c-Jun selectively participates in HPV^−ve^ tumors (panel **b**). The arrowhead indicates the supershifted bands. The intensities of super-shifted bands indicated and quantified in densitometric analysis. NS; non-specific binding.

The composition of functional AP-1 complex in both HPV^+ve^ and HPV^−ve^ tumors was further dissected in band supershift assay with specific antibodies to identify the extent of participation of each AP-1 family proteins. The band shift assay demonstrated, c-Fos and Fra-2 as the major DNA binding partners involved in the formation of functional AP-1 complex with minor involvement of JunD/JunB in HPV^+ve^ well differentiated tumors (64.3%, 9/14) (Fig. 2a & Table 2). In contrast, majority (80.6%, 29/36) of poorly differentiated HPV^−ve^ cases showed minor but consistent involvement of c-Jun along with c-Fos and Fra-2 (Fig. 2b & Table 2). In almost all cases, about 90% of supershifted band was formed by c-Fos and Fra-2 which showed a total supershift making them the chief constituents of functional AP-1 in tongue cancer. Both tongue cancer cell lines also showed similar functional AP-1 complex formation mainly by c-Fos and Fra-2 along with JunB and JunD though they differ in their extent of involvement (Fig. 2c,d). HPV^+ve^ cells however, showed only about 40% involvement of Fra-2 (Fig. 2c) and no involvement of c-Jun in HPV^−ve^ cells (Fig. 2d).

### Differential overexpression of specific AP-1 family proteins in TSCC tissues and cell lines

**Figure 3:**
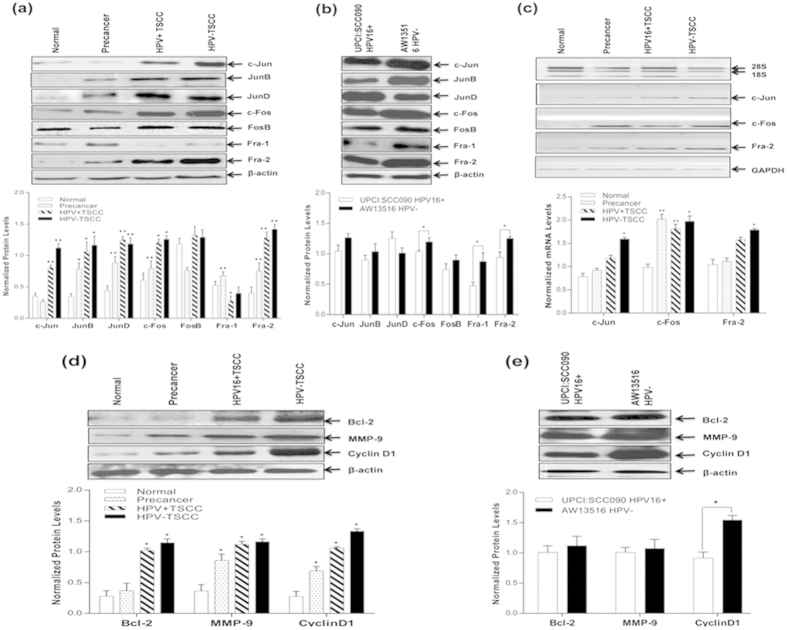
(**a**–**e**): Differential expression pattern of AP-1 family proteins and their target genes in different grades of tongue tissues and cell lines. Representative immunoblots showing overexpression of AP-1 family proteins (c-Jun, JunB, JunD, c-Fos & Fra-2) and their target genes (Bcl-2, MMP-9 and Cyclin D1) in tongue tumor biopsies (**a**) and cell lines (**b**). 40 μg cellular protein from tongue tissues and cell lines were resolved on 10% SDS-PAGE, electrotransferred and probed with specific antibodies against all AP-1 family proteins and their target genes (Bcl-2, MMP-9 and Cyclin D1). To confirm equal loading of protein, the membranes were stripped and re-probed for β-actin expression and quantitation of bands was performed by densitometric analysis (lower panel of **a** & **b**). Note that there is significantly a higher expression of c-Fos & Fra-2 in tumor tissues, while Fra-1 is completely absent. Panel c; RT-PCR showing elevated mRNA expression profile of c-Jun, c-Fos & Fra-2 transcripts in tongue tumor tissues (**c**). GAPDH gene was used as internal loading control (lower panel of **c**). (Panel **d**,**e**); showing overexpression of Bcl-2, MMP-9 and Cyclin D1 genes in HPV^+ve^ and HPV^−ve^ tongue tumor tissues (**d**) and cell lines (**e**). β-actin gene was used as an internal control (lower panels of **d**,**e**). Significant (p < 0.05) when compared with control vs. cases and HPV^+ve^ vs. HPV^−ve^ cell lines. The data in bar diagram are expressed as the mean (±SEM or ±SD) of three independent experiments. *p < 0.05 & **p < 0.01, ***p < 0.001.

Western blotting was performed to study the expression pattern of AP-1 family proteins (c-Jun, JunD, JunB, c-Fos, FosB, Fra-1 and Fra-2) in all spectrum of tongue tissue biopsies from normal, precancer to cancer including TSCC cell lines. AP-1 proteins, c-Jun (60%; 30/50, p = 0.0001), c-Fos (80%; 40/50, p = 0.0001) and Fra-2 (76%; 38/50, p = 0.0001) showed significantly a higher level of expression specifically in majority of HPV^−ve^ tongue cancer cases as compared to precancer and normal controls (Fig. 3a, Table 3). These data very well corroborate the results of band shift assay (Fig. 2a,b). Also, JunB (p = 0.003) and JunD (p = 0.0001) proteins showed elevated expression but Fra-1 showed a low or no expression (Fig. 3a & Table 3) in tumors but it was highly expressed in normal controls (p = 0.0001) (Fig. 3a). Interestingly, despite all AP-1 proteins were gradually overexpressed with the increasing severity of lesions, only a few participated in AP-1 DNA binding activity. When, we differentiated between HPV^+ve^ and HPV^−ve^ tumors, we observed relatively a higher level of c-Jun, c-Fos and Fra-2 expression in HPV^−ve^ tumors as compared to those in HPV^+ve^ TSCCs. Though, other members except Fra-1 showed a higher expression, there was no significant difference with respect to HPV status (Fig. 3a & Table 4). Overall, gradually an increased expression of c-Jun, JunB, JunD, c-Fos and Fra-2 was observed as the lesions progressed towards malignant phenotype. Both the tongue cancer cell lines also showed comparable results but Fra-1, however showed higher expression in both cell lines (Fig. 3b).

### Higher expression of AP-1 family gene transcripts in primary tongue tumors and cell lines

To compare AP-1 family protein expression, the gene transcripts were evaluated in total RNA isolated from tongue tissue biopsies from cancer, precancer and normal controls and tongue cancer cell lines by RT-PCR. As shown in Fig. 3c, the majority of tongue cancer cases showed higher expression of c-Jun, c-Fos and Fra-2 transcripts as compared to precancer and normal controls but the expression level of c-Fos and Fra-2 transcripts well corroborated the results of western blotting (Fig. 3a,b). We also observed similar results with tongue cancer cell lines but the expression of these genes was slightly higher in HPV^−ve^ cells (figures not provided).

### Upregulation of AP-1 target genes; MMP-9, CyclinD1 and Bcl-2 in tongue tumorigenesis

The possible factors that may contribute in potential tumor invasion and metastasis are MMP-9, Bcl-2 and Cyclin D1 genes which are known to be regulated by AP-1. We therefore, examined the expression pattern of these genes in tongue tissue biopsies and cell lines by immunoblotting. As expected significantly a higher expression of MMP-9. Bcl-2 and Cyclin D1 proteins (P < 0.05) (Fig. 3d & Table 3) was observed in the majority of cancer cases as compared to those in precancer and adjacent normal controls. As high as 80% (40/50; P = 0.0001) of tongue cancer cases showed strong expression of MMP-9, 68% (34/50; p = 0.06) for Bcl-2 and 78% (39/50; P = 0.0001) for cyclin D1 genes (Fig. 3d & Table 3). In order to examine the influence of HPV infection on these genes, we categorized them in HPV^+ve^ and HPV^−ve^ tumors. As depicted in the Fig. 3d and Table 4, we observed a slightly higher level of expression of all these genes in HPV^−ve^ tongue tumors. These target genes were also found to be upregulated in both the tumor cell lines but they are more pronounced in HPV^−ve^ cells (Fig. 3e).

### Fra-2 silencing reduced expression of c-Jun, c-Fos, MMP-9 and HPV16 E6/E7 but upregulated p53 in tongue cancer cells

**Figure 4:**
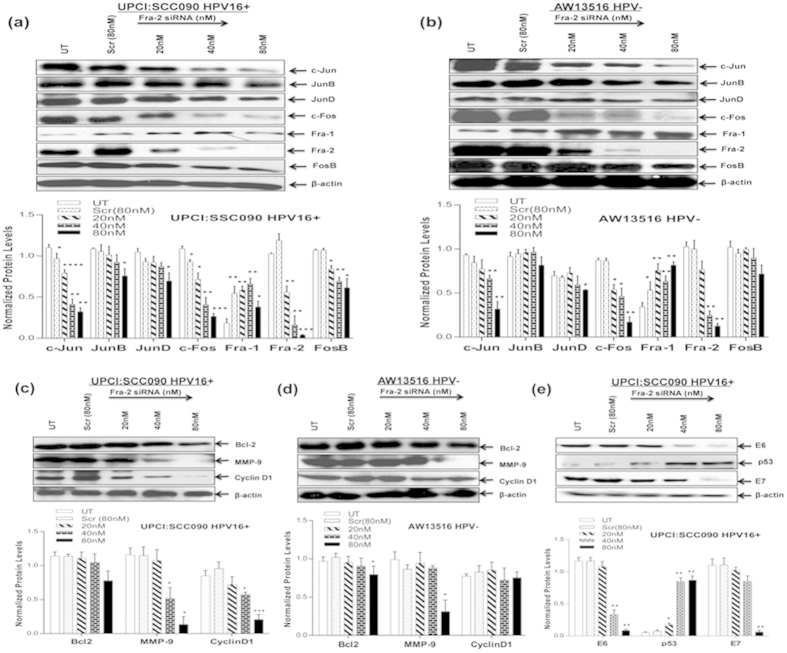
(**a**–**e**): Effect of Fra-2 silencing by specific siRNA in HPV^+ve^ and HPV^−ve^ TSCC cells. Representative immunoblots (Panel **a**,**b**) showing complete inhibition of Fra-2 expression at the concentration of 80 nM in both UPCI:SCC090;HPV16^+ve^ (**a**) and AW13516;HPV^−ve^ cells (**b**). 40 μg cellular proteins isolated from both the cell lines following treatment with Fra-2-siRNA (20 nM-80 nM) and scrambled control siRNA (80 nM) for 48 hours were examined for its effects on AP-1 family proteins (**a**,**b**), their target genes; Bcl-2, MMP-9 and CyclinD1 (**c**,**d**) and HPV16 E6/E7 (**e**). To ensure equal loading of protein, β-actin was used as an internal control. Quantitation of bands was performed by densitometric analysis. Significant (p < 0.05) when compared with untreated control vs. treated group. The data in bar diagram are expressed as the mean ± SD of three independent experiments. *p < 0.05 & **p < 0.01.

Since we observed significantly a higher expression and transactivation of Fra-2 particularly in HPV^−ve^ aggressive tongue cancer tissues and cell lines, we examined the extent of functional contribution of Fra-2 in aggressiveness of the disease by silencing Fra-2 using Fra-2 specific siRNA in both HPV ^+ve^ and HPV^−ve^ tongue cancer cell lines. As presented in the Fig. 4a,b, transfection of these cells by increasing concentration (20 nM to 80 nM) of Fra-2 siRNA resulted in a dose-dependent decline in the expression of Fra-2, while, it remained unchanged in scrambled siRNA treated cells even at the highest concentration (80 nM) used. Inhibition of Fra-2 expression was discernible even at 20 nM and a complete silencing was observed at 80 nM in both the cancer cells (Fig. 4a,b).

Interestingly, Fra-2 downregulation was accompanied by a signaling that led to concomitant reduction of specific AP-1 proteins c-Fos and c-Jun expression which were also upregulated and considered important for aggressive tongue tumorigenesis (Fig. 4a,b). Curiously enough, Fra-1 which was completely absent in TSCCs showed an elevated expression as the concentration of siRNA increased in both the cell lines though it was less pronounced in HPV^+ve^ cells (Fig. 4a,b). Most interestingly, silencing of Fra-2 led to a significant (~95%) downregulation of viral oncogenes E6/E7 expression at 80 nM concentration and a dose-dependent accumulation of p53 level in HPV^+ve^ cells (Fig. 4e). p53 expression was detectable in cells treated with as low as 20 nM and maximum accumulation of p53 was observed at 80 nM. In contrast, untreated/scrambled siRNA treated cells showed very low or negligible expression of p53 (Fig. 4e).

In order to examine the effect of Fra-2 silencing on AP-1 target genes associated with aggressive tumorigenesis, we checked the expression of Bcl-2, MMP-9 and cyclin D1 in siRNA treated tongue cancer cell lines. As shown in Fig. 4c,d, Fra-2 siRNA at the 80 nM concentration resulted in >90% loss of specifically MMP-9 expression while it remained unchanged in cells treated with equi-molar concentration of scrambled siRNA. The expression of cyclin D1 and Bcl-2 remained almost unremarkable in HPV^−ve^ cells (Fig. 4d) but cyclin D1 expression reduced >70% mainly in HPV^+ve^ cells (Fig. 4c). The cancer cells transfected with Fra-2-siRNA (20–80 nM) at 48 hr or prior to harvesting, resulted in only about 30% reduction in cell proliferation (Supplementary Fig. 2a & b).

### Fra-2 silencing reduced invasion and migration of tongue cancer cells *in vitro*

**Figure 5:**
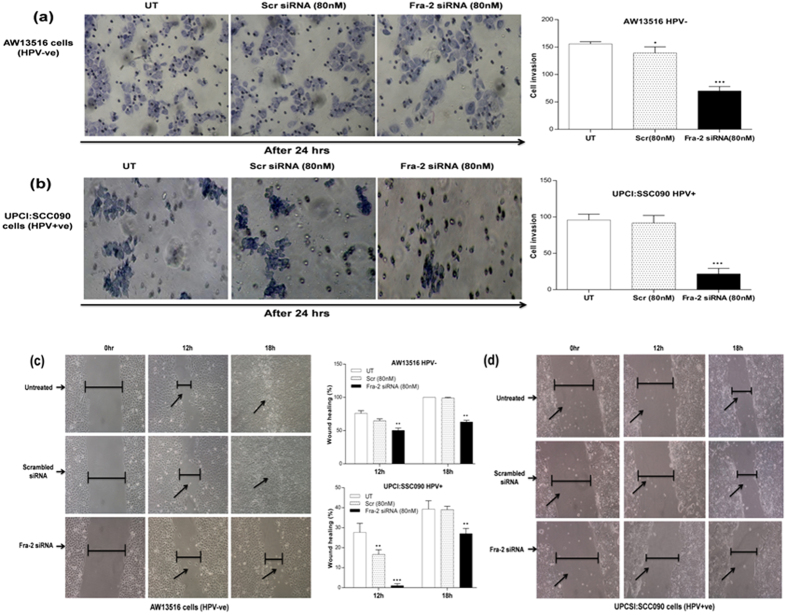
(**a**–**d**): Effect of Fra-2 silencing on cell invasion and migration of HPV^+ve^ and HPV^−ve^ TSCC cells. Fra-2 knockdown by specific siRNA leads to alterations in invasion in both HPV^−ve^ (**a**) and HPV^+ve^ (**b**) TSCC cells. Matrigel invasion assay of untreated, scrambled siRNA and Fra-2 siRNA treated in TSCC cells. Equal number of cells (1 × 10^5^) were seeded in the Matrigel coated upper chamber. After 24 hours of incubation at 37 °C and 5% CO_2_, the cells that invaded through the membrane were fixed and stained with Giemsa, and images were captured using inverted microscope. Representative fields of view for each well are shown. Cell invasion was quantified by counting cells in six random fields. (**c**,**d**) Scratch assay of Fra-2 silencing demonstrated that cell migration was dramatically decreased in experimental groups after Fra-2 transfection compared with untreated and scrambled treated control groups. Inverted microscope images of wound closure at 0 hours, 12 hours and 18 hours in AW13516 (**c**) and UPCI:SCC90 (**d**) cells as indicated. Migration rate was calculated by image J software. The data in bar diagram are expressed as the mean ± SD of three independent experiments. *p < 0.05, **p < 0.01, ***p < 0.001 & ****p < 0.0001.

For evaluation of the potential effects of Fra-2 silencing on the invasive properties of both HPV^+ve^ and HPV^−ve^ tongue cancer cell lines, we have employed Matrigel invasion assay (Fig. 5a,b) using standard procedure^24^. The staining of invading cells through the polycarbonate membranes demonstrated that the invasion of cells was reduced after Fra-2 siRNA silencing when compared with invasion of untreated or scrambled siRNA treated cells (Fig. 5a,b). Transfection with Fra-2 siRNA resulted in a remarkable decrease in the number of invading cells in both HPV^+ve^ and HPV^−ve^ cells. Interestingly, Fra-2 silencing was more effective in HPV^+ve^ cells to prevent cell invasion through the Matrigel as compared with HPV^−ve^ cells. Quantitation of the invading HPV^−ve^ cells showed only 45% (±70 cells, p = 0.001) invasion after treatment with Fra-2 siRNA when compared to untreated (±155.6 cells; 100%) or scrambled siRNA treated cells (±139.5 cells; 90%) (Fig. 5a). In HPV16^+ve^ cells, we observed only 28% (±21.66 cells, p = 0.008) invasion after siRNA treatment as compared to untreated (±95.6 cells) or scrambled treated groups (±91.6 cells; 91%) (Fig. 5b). The untreated groups served as negative control and scrambled siRNA treated was considered as treated controls for both the cell lines. To determine the effects of Fra-2 on migratory property of TSCC cells and its ability to accelerate the closure of a wound, scratch assay was performed. Fra-2 knockdown in AW13516 (HPV^−ve^) cells for 18 hours showed an ~63% migration rate while untreated or scrambled treated control cells showed ~100% migration (Fig. 5c). Interestingly, in HPV16^+ve^ cells following Fra-2 siRNA treatment for 18 hours showed only ~27% migration while ~40% and ~39% migration was observed in untreated or scrambled siRNA treated cells respectively (Fig. 5d). These results together correlate well with the significant decrease in proinvasive factor MMP-9 expression and the reduction in cell invasion and migration after knocking down Fra-2 in both HPV^+ve^ and HPV^−ve^ tongue cancer cells.

## Discussion

Human oral tongue cancer is a most aggressive disease and generally associated with a very high invasive and metastatic potential. Transcription factor, AP-1 plays a critical role in regulating wide variety of cellular processes including cell differentiation, apoptosis, transformation and signal transduction pathways, specifically during progression and metastasis of several cancers including oral cancer^15^,^19^,^20^,^22^. AP-1 has therefore emerged as an important drug discovery target but its role in tongue cancer has not been studied.

Since high-risk HPV type 16 is a major oncogenic virus often associated with tongue cancer and its oncogenic expression is again controlled by AP-1 and its family proteins, we have molecularly dissected the role of AP-1 and its family proteins and HPV during tongue tumerigenesis. We have also examined the role of AP-1 downstream target genes Bcl-2, MMP-9 and Cyclin D1 in tongue cancer.

The prevalence of HPV infection in TSCCs has been reported to vary between 0 to as high as 82%, depending on the geographic regions, tumor sites and techniques applied for detection of the virus^6^,^7^,^26^,^27^,^28^. Till date there have been only two studies^7^,^26^ from India which showed contrasting report of HPV prevalence with 48% vs. 82% in TSCCs. In the present study, we observed 28% prevalence (14/50) of HR-HPV16 which was found to be the exclusive type (100%; 14/14) and more than 90% of them were present in an integrated form (to be published elsewhere). Our results corroborate earlier studies that showed predominance of HPV type 16 (100%) in oral tongue cancer particularly in the base of tongue (85.7%) except one study that reported a very high prevalence (82%) of HPV that too of HR-HPV type 18^26^. The reasons for such a contrasting result are not known. It is interesting to note that the majority of HPV16^+ve^ cases belonged to well differentiated tumors (78.6%) which are expected to show better prognosis when treated. This was demonstrated first in oral cancer^11^ and later in other head and neck cancers^9^,^29^,^30^,^31^,^32^. It was further demonstrated that HPV infection promotes selective participation of p65 in NF-κB complex formation leading to overexpression of p21 that induces well differentiation and better prognosis of oral cancers^11^.

When we correlated HPV positivity with the tumor location, majority (85.7%) of HPV infection was found to be located in the base of the tongue and are most aggressive than those located in the mobile tongue or other sites of the tongue (14.3%). Our data are in good agreement with earlier studies which showed HPV16 as the most predominant type specifically found at the base of the tongue^6^,^28^,^33^. Observations of a very high prevalence of HPV infection specifically at the base of the tongue is possibly due to its secluded location that allows minimal exposure to tobacco insults and food intake etc. It is intriguing to note that inspite of smoking being strongly linked to HNSCC and HR-HPV being an yet another important causative factor associated with TSCC, smoking appears to be protective against oral HPV infection. This is clear from our results that out of 10 female TSCC patients, 80% (8/10) were HPV^+ve^ and 75% (6/8) of them were nonsmokers. Similarly, out of 40 male TSCC patients, all were smokers but only 15% (6/40) of them were HPV^+ve^. It suggests that female patients who are generally non-smokers or mild smokers in India and are more likely to contract HPV infection leading to well differentiated tumors which would show better prognosis while males, in contrast, are often heavy smokers and are likely to have HPV^−ve^ poorly differentiated tumors with worst prognosis. It gains support from recent studies^34^,^35^ which also demonstrated significantly a lower rate of HPV infection in smokers as compared to nonsmokers. Thus, it appears that nicotine released and oxidative stress created in mouth due to smoking may inhibit HPV infection and its persistence.

A strong DNA binding activity of AP-1 observed in tongue cancer cases as compared to precancer or normal adjacent controls corroborate several earlier studies in variety of other carcinomas including oral and esophageal carcinomas^15^,^19^,^20^,^22^,^36^,^37^. It is known that the expression of HPV16 oncoproteins E6 and E7 can induce AP-1 activity and are responsible for tumorigenic transformation but at the same time, the expression of these viral oncoproteins is dependent mainly on the availability of AP-1 which acts as a signaling epicenter for HPV-induced tumorigenesis^22^,^38^,^39^. The band supershift assays demonstrated an altered functional AP-1 complex formed by heterodimerization between c-Fos and Fra-2 instead of canonical partners, c-Fos and c-Jun in both HPV^+ve^ and HPV^−ve^ tongue carcinoma. In sharp contrast to almost all other carcinomas, preferential heterodimerization between c-Fos and Fra-2, both of which are known to have highly oncogenic potential^24^,^25^, appears to make tongue carcinoma most aggressive.

Interestingly, in majority of HPV^−ve^ tongue cancer cases which are shown to be highly aggressive and have worst prognosis, there is a selective participation of c-Jun along with c-Fos/Fra-2 heterodimer mainly in poorly differentiated tumors (PDSCCs/MDSCCs). Furthermore, Fra-2 silencing also downregulates c-Jun. It is indicative of a crosstalk between c-Jun and Fra-2/c-Fos that makes this subtype of tongue cancer highly aggressive. It gains support from recent report of Mariani *et al.* (2007) who demonstrated amplification and overexpression of c-Jun can block adipocytic differentiation leading to undifferentiated highly aggressive tumors^40^. Zhou *et al.* (2013) also showed that changes in BRCA1 gene expression is regulated by c-Jun and Fra-2 that bind to the CRE element in early stages of ovarian carcinoma that suppress BRCA1 expression in sporadic tumors and promote tumor progression^41^. Together, these findings suggest that selective participation of c-Jun with c-Fos-Fra-2 protein complex of AP-1 plays an important role in maintaining undifferentiated tumor phenotype, aggressiveness and poor prognosis that may lead to drug resistance, treatment failure and disease relapse.

On the other hand, participation of JunD observed specifically in HPV16^+ve^ cases appears to induce tumor differentiation as majority of the HPV^+ve^ cases were found to have well differentiated tumors (WDSCCs) that show better prognosis. It gains support from the study by Natio *et al.* (2005) who showed that JunD protein plays an important role in bone formation by stimulating differentiation in osteoblasts^42^. Earlier, we have demonstrated a constitutively active AP-1 formed mainly by c-Fos/JunD heterodimers in majority of oral cancers^20^. Interestingly, JunD/JunD homodimer often observed in precancerous oral lesions appears to prevent progression to cancer, but its participation with c-Fos induced lesions to progress to invasive cancer^20^. It has been also suggested that the functional change of JunD from growth suppressor to a growth promoter could be possibly due to presence of mutant form of JunD^43^.

Both HPV^+ve^ and HPV^−ve^ tongue cancer cell lines also provided similar results except that it showed preferential participation of JunB instead of c-Jun observed in HPV negative tongue cancer tissues. Ohyama *et al.* (2008) reported that overexpression of JunB along with c-Fos can enhance 4Nitroquinoline 1-oxide-induced tongue carcinogenesis in rat indicating an important role of JunB/c-Fos crosstalk in tongue cancer^44^. A higher participation of JunD only in HPV16^+ve^ cells indicates that JunD facilitates tumor differentiation leading to better prognosis.

Immunoblotting analysis revealed a very high expression of c-Jun, c-Fos and Fra-2 in almost all tongue cancer cases and cell lines and overexpression of these genes has been shown to contribute to more aggressive disease phenotype and metastasis in breast, oral and other cancers^45^,^46^,^47^. Overexpression of Fra-2 has been shown to induce aggressive progression and metastasis particularly in triple negative breast cancer^24^,^25^. Interestingly, Fra-1 has been demonstrated as oncogene by many authors but we have consistently observed its role as a tumor suppressor as it did not express in cervical, oral, esophageal^18^,^20^,^22^ and ovarian tumor tissues^23^. In contrast, in breast cancer specifically those were ER/PR/Her2 negative, Fra-1 showed significantly a higher expression^48^. Fra-1 is also highly expressed in many other cancers and plays a role in transformation, cell proliferation and metastasis^49^,^50^. Even in the present study, while Fra-1 is almost nil in fresh tongue tumor tissues, it however, shows higher expression in tongue cancer cell lines (Fig. 3a,b). Together these findings suggest that the oncogenic or tumor suppressor function of Fra-1 is dependent on the type of tumor. It is also highly interesting to note that induction of Fra-1 expression in those tumors that do not express Fra-1 can render them chemo-radio-sensitive (Das *et al.* unpublished observation). This gains strong support from our present findings that knocking down of Fra-2 (responsible for aggressive tumorigenesis and drug resistance) led to upregulation of p53 (Fig. 4e) and Fra-1. When we correlated AP-1 family protein expression with HPV status, HPV^−ve^ tumors showed higher expression of c-Jun along with c-Fos and Fra-2 as compared to those in HPV^+ve^ cases, indicating aggressive phenotype of HPV^−ve^ tumors than HPV positives.

The aggressiveness of TSCCs is primarily dependent on its ability to invade adjacent tissues and metastasize to distant organ sites and the most known factors responsible are MMP-9, Cyclin D1 and Bcl-2 which also have an AP-1 binding site in their promoters. We observed significantly a higher expression of MMP-9, cyclin D1 and Bcl-2 genes in majority of tongue cancer cases and cancer cell lines particularly in HPV^−ve^ cases that show aggressive tumor and worst prognosis. It corroborates several earlier findings^51^,^52^,^53^. It has been also reported that overexpression of c-Jun induces expression of MMP-9 and ECM invasion in tumor cells^54^. It is interesting to note that EGFR which is generally overexpressed in head and neck cancer can induce expression of MMP-9 and leads to the activation of the Raf-MEK-ERK signal transduction pathways which in turn can activate the c-Jun transcription factor. This may also contribute in regulating other critical genes, which are implicated in cell proliferation, differentiation and apoptosis^15^,^55^.

In order to understand the role of Fra-2 in tongue tumorigenesis and its possible cross talk with other critical genes such as c-Jun, c-Fos and MMP-9 that appears to contribute to the aggressiveness of the disease, Fra-2 was silenced by specific siRNA. The results demonstrated complete abolition of Fra-2 expression but expression of all the three critical genes eg. c-Fos, c-Jun and MMP-9 were also downregulated along with Fra-2. Also, HPV E6/E7 showed significant down regulation. Most interestingly, p53 as well as Fra-1 showed an elevated expression. Our observations thus suggest that complete silencing of Fra-2 also inhibits expression of c-Jun, c-Fos and MMP-9 responsible for aggressive tumor behavior but induces expression of p53 and Fra-1 which appears to function as tumor suppressors and may chemo-radio sensitize tongue tumor cells. It provides a clue for development of a novel therapeutic approach for effective targeting of chemo-radio resistant tongue cancer stem cells that cause cancer relapse. Our functional *in vitro* cell invasion and migration assays demonstrated Fra-2 knockdown can significantly reduce TSCC cell invasion and migration capability of both HPV^+ve^ and HPV^−ve^ tongue cancer cells *in vitro*. It has previously been reported that Fra-2 silencing significantly decreased cell migration and invasion in breast cancer cells^24^. These results together suggest potential effects of Fra-2 silencing on inhibiting invasion, migration and chemo-radio sensitization of TSCC cells.

In conclusion, our findings suggest that HR-HPV type 16 predominantly infects base of the tongue and persists preferably in nonsmoking individuals and is responsible for a major proportion of well differentiated tongue cancers that show better prognosis when treated. Higher DNA binding and overexpression of c-Fos and Fra-2 which form the functional AP-1 complex increased as a function of severity of tongue lesions, yet selective participation of c-Jun promotes poor differentiation, aggressive tumorigenesis and worst prognosis only in HPV^−ve^ cases. Nevertheless, Fra-2 appears to play a critical role in tongue tumorigenesis since selective silencing of Fra-2 led to downregulation of tumorigenic genes; c-Fos, c-Jun, MMP-9 and HPV E6/E7 but upregulation of p53 and Fra-1 that may chemo-radio-sensitize tongue cancer cells leading to better treatment outcome.

## Methods

### Study subjects and collection of clinical samples

A total of 100 fresh tongue tissue biopsies of different histopathological grades comprising precancer (n = 20), cancer (n = 50) and adjacent normal controls (n = 30) were collected from the Department of ENT Surgery of Dr. Ram Manohar Lohia Hospital, New Delhi. None of these patients received any pre-operative radiation and chemotherapy. The clinico-pathological and epidemiological details of the patients were collected using standard proforma. Biopsy samples obtained immediately after surgery in sterile vials containing cold phosphate buffer saline (PBS), transported to lab on ice and stored in −80 °C deep freezer till further analysis.

The study was approved by the Institutional Ethics Committee of Dr. B. R. Ambedker Center for Biomedical Research (ACBR), University of Delhi, Delhi (India) and Dr. Ram Manohar Lohia Hospital, New Delhi. Written informed consent was obtained from all the subjects prior to their inclusion in the study. The study was carried out in accordance with the guidelines and principles of the Helsinki Declaration.

### Cell lines and cell culture

The two tongue cancer cell lines; one HPV16 positive, UPCI:SCC090 (a kind gift from Dr. Susanne M. Gollin, University of Pittsburgh, Pittsburgh, PA) was derived from the base of tongue squamous cell carcinoma^56^ and the other HPV negative cell line, AW13516 (a kind gift from Dr. M. M. Vaidya, ACTREC, Tata Memorial Hospital, Navi Mumbai, India) derived from the squamous cell carcinoma of human tongue^57^, were maintained in MEM and IMDM medium respectively, supplemented with 10% FBS (Gibco, Invitrogen, CA) and antibiotics (penicillin and streptomycin) at 37 °C and 5% CO_2_.

### DNA extraction and HPV genotyping

High molecular-weight genomic DNA was isolated from precancer, cancer and adjacent normal tissues as controls and two tongue cancer cell lines by standard procedure of proteinase K digestion, phenol-chloroform extraction and ethanol precipitation as followed in the lab. The initial HPV diagnosis was performed by using a pair of consensus degenerate primers (MY09 and MY11) derived from the highly conserved L1 region of HPV genome (Supplementary Table 1). PCR was performed in a 25 μl reaction mix containing 100 ng DNA, 10 mM Tris-HCl (pH 8.4), 50 mM KCl, 1.5 mM MgCl_2_, 125 μM of each dNTP (dATP, dCTP, dGTP and dTTP), 5 pmol of each oligonucleotide primer and 0.5 U Taq DNA polymerase (Bangalore Genei, Bangalore, India). β-globin gene was used as an internal control. HPV positive samples were subjected to comprehensive HPV genotyping assay using type-specific PCR and PGMY-reverse line blot assay (RLB) as per recommended protocol provided under the WHO HPV LabNet program^58^. Although the HPV^+ve^ cell lines have previously been characterized, we reconfirmed the HPV status in these cell lines by PCR.

### Preparation of protein extract and electrophoretic mobility shift assay (EMSA)

Nuclear extracts from all spectrum of tongue tissue biopsies and cell lines (UPCI:SCC90 and AW13516) were prepared by the method of Dignam^59^ with minor modification as described earlier^11^. The concentration of nuclear protein extracts was determined spectrophotometrically using standard Bradford method (Bio-Rad laboratories, Inc. CA) and stored at −80 °C till further use. AP-1-specific DNA binding activity in the nuclear extracts from fresh tongue tissues and cell lines was examined by EMSA using consensus oligonucleotide of AP-1 (5′-CGCTTGATGACTCAGCCGGAA-3′) as described earlier^20^. Oct-1 consensus oligonucleotide (5′-TGTCGAATGCAAATCACTAGAA-3′) was used as a control. The above oligonucleotides were annealed and labelled with [γ^**32**^P] ATP (3000 Ci/mmol; Jonaki, Hyderabad, India) by T4 polynucleotide kinase and gel purified in a 15% polyacrylamide gel^17^. Briefly, the binding reaction was performed in a 25 μl reaction volume containing 50% (v/v) glycerol, 60 mM HEPES (pH 7.9), 20 mM Tris-HCl (pH 7.9), 300 mM KCl, 5 mM EDTA, 5 mM DTT, 100 μg of BSA per ml, 2.5 μg of poly-dI-dC and 10 μg of nuclear extract. After 5 min, 10,000 cpm of the [γ^**32**^P] ATP 5′-end labelled double-stranded AP-1 oligonucleotide probe was added and the incubation was continued for additional 25 min at room temperature and the DNA-protein complex was resolved in a 4.5% non-denaturing polyacrylamide gel. The gel was then dried and visualized by Phosphorimager (Fujifilm FLA-5100) using Multi Gauge-ver 3.X anlaysis software. For monitoring composition of AP-1 and Oct-1, following antibodies from Santa Cruz Biotechnology (Santa Cruz, CA) were used: c-Jun (sc-45), JunB (sc-73), JunD (sc-74), c-Fos (sc-253), FosB (sc-48), Fra-1 (sc-605), Fra-2 (sc-171). The quantitative densitometric analysis was performed using Alpha Ease FC version 4.1 (Alpha Innotech Corporation, IL).

### Western blotting

Protein extracted from tumor tissues and cell lines (40 μg/lane) were separated on 8–12% SDS-PAGE, electrotransferred to PVDF membrane and immunodetected using respective antibodies as described earlier^20^. The membrane was blocked with 10% fat free milk and incubated overnight in PBS with 5% milk, 0.05% Tween-20 and probed with polyclonal rabbit primary antibodies of respective AP-1 family members at 4 °C. These blots were washed, incubated with HRP- tagged anti-rabbit IgG secondary antibodies and visualized by Luminol detection kit (Santa Cruz Biotech, USA). The membranes were re-probed for β-actin expression as an internal control. The quantitative analysis of the bands was done using Alpha Ease FC version 4.1 (Alpha Innotech Corporation, IL). The level of expression of proteins was quantitated on an arbitrary scale with respect to β-actin expression as strong (++++)  =  >50%; Medium (++)  = 10–50%; Weak (+)  =  <10% of β-actin expression and Nil/not detectable (−) = <1 as described earlier^20^.

### RNA extraction and RT-PCR

Total RNA was isolated from fresh tongue biopsies comprising pre-cancer, cancer and normal adjacent control tissues and two tongue cancer cell lines using TRI reagent as per manufacturer’s protocol (Sigma-Aldrich Inc, USA). The quality and integrity of RNA was checked spectrophotometrically in a Nanodrop (Thermo scientific,USA) and on ethidium bromide-stained 1% agarose gel. For reverse transcription-PCR (RT-PCR) analysis, the isolated RNA (2 μg) was employed to prepare cDNA using first strand cDNA synthesis kit (Fermentas, USA). RT-PCR reactions were carried out in duplicates using AP-1 specific primers. The primers used for RT-PCR are listed in Supplementary Table 2. The mRNA expression levels of AP-1 genes were quantitated and normalized by GAPDH used as an internal loading control. The PCR products were analyzed on 2% agarose gel electrophoresis.

### siRNA transfection and interference assay

For Fra-2-siRNA interference assay, transient transfection of commercially available Fra-2 siRNA oligonucleotides (Santa Cruz, biotech, USA) was performed according to the manufacturer’s protocol with some minor modifications^60^. Briefly, the complexes of Fra-2-siRNA-RNAimax (Invitrogen, USA) of different concentrations (20 nM, 40 nM and 80 nM) or scrambled siRNA (80 nM) were prepared in 6 wells plates in triplicates, after which 1 × 10^5^ cells/well (AW13516 & UPCI:SCC090) and 2 ml antibiotics-free normal growth medium supplemented with 10% FBS was added and incubated at 37 °C in a CO_2_ incubator for 24–48 hours until the cells were 40–60% confluent followed by microscopic examination and protein expression analysis by immunoblotting.

### *In vitro* Matrigel cell invasion assay

The effect of Fra-2 silencing on invasive characteristics of tongue cancer cells was evaluated using Matrigel invasion assay. After 24 hours of Fra-2 siRNA or scramble siRNA transfection, TSCC cells were trypsinized and resuspended in FBS-free medium. A total of 1 × 10^5^ cells were plated in upper chamber of the transwell with a matrigel-coated membrane (8.0 μm pore size; Corning Costar Corp. USA). Medium with serum was added to the lower chamber as a chemoattractant. After incubation for 24 hours, cells on the lower surface of the membrane were fixed with formaldehyde and stained with Giemsa. Non-migratated cells were mechanically removed by a cotton swab. The images of invaded cells were acquired by an inverted microscope with a magnification of 20X. The number of invaded cells was quantified by counting cells in five randomly selected fields under microscope. Comparisons were made between the untreated or scramble siRNA treated and Fra-2 knockdown wells. All experiments were performed in triplicate.

### *In vitro* cell migration assay

To investigate the migratory capacity of TSCC cells, cell scratch assay was performed. Both HPV^+ve^ and HPV^−ve^ TSCC cells were transfected with 80 nM Fra-2 siRNA and scramble siRNA as control as described above and incubated under standard conditions to achieve knockdown of Fra-2. After 24 hours, a scratch was carefully made by scraping through each well using a sterile pipette tip. The cells were then washed with PBS to remove detached cells. Scratches were monitored with an inverted microscope immediately after wounding and after incubation in an incubator (37 °C, 5% CO2) at different time points. Images were taken exactly at the same position before and after the incubation and migration rate was calculated by using image J software.

### Statistical Analysis

The data analysis was performed using the statistical software Graph Pad Prism (version 6.0) and image J software. The association between HPV infection and expression profile of AP-1 proteins among different histopathological grades and clinico-pathological parameters in TSCC cases was determined using Fischer’s exact test and students t-test (two tailed). The p value ≤ 0.05 was considered as statistically significant.

## Additional Information

**How to cite this article**: Gupta, S. *et al.* Selective participation of c-Jun with Fra-2/c-Fos promotes aggressive tumor phenotypes and poor prognosis in tongue cancer. *Sci. Rep.* **5**, 16811; doi: 10.1038/srep16811 (2015).

## Supplementary Material

Supplementary Information

## Figures and Tables

**Table 1:** Clinico-pathological and demographic characteristics and their correlation with HPV16 infection in tongue cancer patients

Characteristics		No. of cases (%)	No. of HPV positive	No. of HPV Negative	*p-value*
Adjacent normal controls		30	nil	—	—
**Precancer (n** = **20)**	**36.7** ± **5.99**	**Mean age** ± **SD**	**nil**	—	
Precancer (n = 20)	Leukoplakia/Erthroplakia	11 (55%)	nil	—	—
	Dysplasia	9 (45%)	nil	—	—
**Cancer (n** = **50)**	**40.48** ± **12.46**	**Mean age** ± **SD**	**14 (28%)**	**36 (72%)**	
**Age range**	<35	30 (60%)	10 (71.4%)	20 (55.6%)	0.3 (ns)
	>35	20 (40%)	4 (28.6%)	16 (44.4%)	
**Gender**	**Male**	**40** (**80%)**	**6** (**42.9%)**	**34**(**94.4%)**	**0.0002**
	Female	10 (20%)	8 (57.1%)	2(5.6%)	
**Religion**	Hindu	40 (80%)	13 (92.9%)	27 (75%)	0.2 (ns)
	Muslim	10 (20%)	1 (7.1%)	9 (25%)	
**Addiction habit (smoking & chewing)**	**Smokers**	42 (84%)	**8** (**57.1%)**	**34**(**94.4%)**	**0.003**
	Non-smokers	**8** (**16%)**	6 (42.9%)	2(5.6%)	
**Tumor site**	**Base of tongue**	22 (44%)	**12** (**85.7%)**	10 (27.8%)	**0.0003**
	Mobile tongue & other sites of tongue	28 (56%)	2 (14.3%)	26 (72.2%)	
**Differentiation status**	**WDSCC**	15 (30%)	**11** (**78.6%)**	4(11.2%)	**0.0001**
	MDSCC	8 (16%)	2 (14.3%)	6 (16.7%)	
	PDSCC	27 (54%)	1 (7.1%)	26 (72.2%)	
**Tumor status**	**T1-T2**	20 (40%)	**11** (**78.6%)**	9 (25%)	**0.0009**
	T3-T4	30 (60%)	3 (21.4%)	27 (75%)	
**Node Status**	**N0-N1**	39 (78%)	**13** (**92.9%)**	26 (72.2%)	0.2 (ns)
	N2-N3	11(22%)	1 (7.1%)	10 (27.8%)	
**Clinical Staging**	Stage I–II	14 (28%)	10 (71.4%)	4 (11.1%)	**0.0001**
	Stage III–IV	36 (72%)	4 (28.6%)	32 (88.9%)	

#n: number of patients. T: primary tumor. N: regional lymph node. Subjects comprised normal controls, precancer and HPV^−ve^ and HPV16^+ve^ cancer patients. P-values were obtained by probability Fisher’s (exact) test using Graph pad Prism 6.0. P-values in bold **(≤0.05)** are considered as statistically significant.

**Table 2:** Cumulative data of activation of AP-1 family proteins in HPV16^+ve^ & HPV^−ve^ tongue cancer cases as observed by band supershift assay.

AP-1 family proteins(↓)	TSCC (n = 50)								*p-value*
	HPV16 positive TSCC (n = 14)				HPV negative TSCC (n = 36)				
	Nil -	Weak +	Moderate ++	Strong ++++	Nil -	Weak +	Moderate ++	Strong ++++	
**c-Jun**	14	—	—	—	7	9	20	—	**0.0001**
**JunB**	9	5	—	—	36	—	—	—	1.0 (ns)
**JunD**	5	3	6	—	36	—	—	—	**0.0001**
**c-Fos**	—	—	2	12	—	1	6	29	1.0 (ns)
**FosB**	14	—	—	—	36	—	—	—	1.0 (ns)
**Fra-1**	14	—	—	—	36	—	—	—	1.0 (ns)
**Fra-2**	—	2	3	9	—	2	2	32	0.31 (ns)

#Data represent DNA binding activity of different AP-1 family proteins in >90% of cases with minor changes in few cases. Arbitrary level of DNA binding in band supershift assay: Nil/not detectable (0%, −); Weak (~25%, +); Moderate (~50%, ++); Strong (>80%, ++++). P-value obtained by probability Fisher’s (exact) test using Graph pad Instat (6.0) comparing the DNA binding of AP-1 proteins (nil+weak vs moderate+strong) among HPV16^+ve^ cancer vs. HPV^−ve^ cancer. P-values in bold (≤0.05) are considered as statistically significant. ns = not significant.

**Table 3:** Cumulative expression pattern of AP-1 family proteins and their downstream gene products in precancer and cancer of the tongue and normal adjacent tongue tissue as observed by immunoblotting.

*Target Proteins*(↓) Expression Level (→)	*Normal adjacent controls (n* = *30)*				*Precancer (n* = *20)*				*Tongue cancer (n* = *50)*				*p-value*
	Nil (−)	Weak (+)	Moderate (++)	Strong (++++)	Nil (−)	Weak (+)	Moderate (++)	Strong (++++)	Nil (−)	Weak (+)	Moderate (++)	Strong (++++)	
**c-Jun**	6	14	10	—	—	10	8	2	—	4	16	30	0.2 (ns)^1^, **0.0001**^**2**^, **0.0002**^**3**^
**JunB**	2	10	15	3	2	5	10	3	2	3	20	25	0.7 (ns)^1^**, 0.003**^2^**, 0.006**^3^
**JunD**	5	15	10	—	1	4	12	3	1	5	12	32	**0.008**^1^**, 0.0001**^2^, 0.4 (ns)^3^
**c-Fos**	2	20	8	—	—	2	10	8	—	—	10	40	**0.0001**^1^**, 0.0001**^**2**^, 0.07^3^
**FosB**	4	9	14	3	—	5	10	5	2	8	10	30	0.2 (ns)^1^**, 0.04**^2^, 0.7 (ns)^3^
**Fra-1**	—	5	10	15	1	4	10	5	27	14	7	2	0.4 (ns)^1^**, 0.0001**^2^**, 0.0001**^3^
**Fra-2**	5	20	5	—	—	2	10	8	—	—	12	38	**0.0001**^1^**,0.0001**^2^, 0.07^3^
**Bcl-2**	4	18	8	—	—	5	9	6	—	1	15	34	0.4 (ns)^1^, 0.06^2^**, 0.006**^3^
**MMP-9**	6	10	12	2	—	2	15	3	—	—	10	40	**0.002**^1^**, 0.0001**^2^, 0.07^3^
**CyclinD1**	7	12	10	1	3	4	9	4	1	3	7	39	**0.008**^1^**, 0.0001**^2^**, 0.009**^**3**^

#Arbitrary level of expression as detected by immunoblotting: Nil/not detectable (−); Weak (+); Moderate (++); Strong (++++). P-value obtained by probability Fisher’s (exact) test using Graph pad Instat 6.0 comparing the expression of proteins (nil + weak vs. moderate + strong) among ^1^Control vs. Pre-cancer, ^2^Control vs. Cancer, ^3^Pre-cancer vs. Cancer. P-values in bold (≤0.05) are considered as statistically significant. ns = not significant.

**Table 4:** Cumulative expression patterns of AP-1 family proteins and its downstream genes in HPV16^+ve^ and HPV^−ve^ tongue cancer cases.

*Target proteins*(↓) Expression Level (→)	TSCC (n = 50)								*p-value*
	*HPV16 positive TSCC (n* = *14)*				*HPV negative TSCC (n* = *36)*				
	Nil −	Weak +	Moderate ++	Strong ++++	Nil −	Weak +	Moderate ++	Strong ++++	
**c-Jun**	—	3	6	5	—	1	10	25	0.06
**JunB**	2	2	4	6	—	1	16	19	**0.01**
**JunD**	—	2	2	10	1	3	10	22	1.0 (ns)
**c-Fos**	—	—	3	11	—	—	7	29	1.0 (ns)
**FosB**	—	3	4	7	2	5	6	23	1.0 (ns)
**Fra-1**	6	7	1	—	21	7	6	2	0.4
**Fra-2**	—	—	2	12	—	—	10	26	1.0 (ns)
**Bcl-2**	—	1	5	8	—	—	10	26	0.2
**MMP-9**	—	—	6	8	—	—	4	32	1.0 (ns)
**Cyclin D1**	1	3	2	8	—	—	5	31	**0.004**

#Arbitrary level of expression as detected by immunoblotting: Nil/not detectable **(−)**; Weak (+); Moderate (++); Strong (++++). P-value obtained by probability Fisher’s (exact) test using Graph pad Instat 6.0 comparing the expression of proteins (nil + weak vs. moderate + strong) among HPV16 ^+ ve^ Cancer vs. HPV^−ve^ Cancer. P-values in bold (≤0.05) are considered as statistically significant. ns = not significant.
