# Supplementary material for: Selective participation of c-Jun with Fra-2/c-Fos promotes aggressive tumor phenotypes and poor prognosis in tongue cancer
Source: Sci Rep. 2015 Nov 19;5:16811. doi: 10.1038/srep16811 (PMC4652185; doi:10.1038/srep16811)
Supplement: Supplementary Information [file srep16811-s1.pdf]

Supplementary Information

Selective participation of c-Jun with Fra-2/c-Fos promotes aggressive tumor phenotypes and poor prognosis in tongue cancer

Shilpi Gupta[1*], Prabhat Kumar[1,2*], Harsimrut Kaur[1], Nishi Sharma[3], Daman Saluja[1], Alok C. Bharti[4] & Bhudev C. Das[1,2***]**

[1]Department of Molecular Oncology, Dr. B.R. Ambedkar Centre for Biomedical Research (ACBR), University of Delhi, New Delhi-110007, India.

[2]Amity Institute of Molecular Medicine & Stem Cell Research (AIMMSCR), Amity University Campus, Uttar Pradesh, Sector-125, Noida-201313, India.

[3]Department of Otorhinolaryngology, Post Graduate Institute of Medical Education and Research, Dr. Ram Manohar Lohia (RML) Hospital, New Delhi-110010, India.

[4]Division of Molecular Oncology, Institute of Cytology & Preventive Oncology (ICMR), Uttar Pradesh, Sector-39, Noida-201301, India.

Running Title: Role of Fra-2/c-Fos in tongue cancer

Bhudev Das (*Corresponding Author)**
Molecular Oncology Laboratory,
B.R. Ambedkar Centre for Biomedical Research (ACBR),
University of Delhi, New Delhi, 110007, India
Tel: +91-0120-4586855, Mob: +919810566870, +919810537835
E-Mail:- bcdas48@hotmail.com, bcdas@amity.edu

Dr. Alok C. Bharti (Co-Corresponding Author)**
Division of Molecular Oncology,
Institute of Cytology & Preventive Oncology (ICMR), Noida, India
E-Mail:- bhartiac@icmr.org.in

***Equally contributed to first authorship**

Supplementary Figures

Supplementary Figure S1: Detection of HPV genotypes by RLB assay in TSCCs. Representative RLB assay showing specificity of the assay for identification of different HR and LR-HPV genotypes. HPV reference types showing presence of indicated HPV types in particular lane (left panel). PGMY-RLB showing presence of exclusively the HPV type 16 in TSCCs (Right panel). Lane 1 to 10, HPV16 positive TSCC cases; Lane PC, positive control (UPCI:SCC090 genomic DNA).

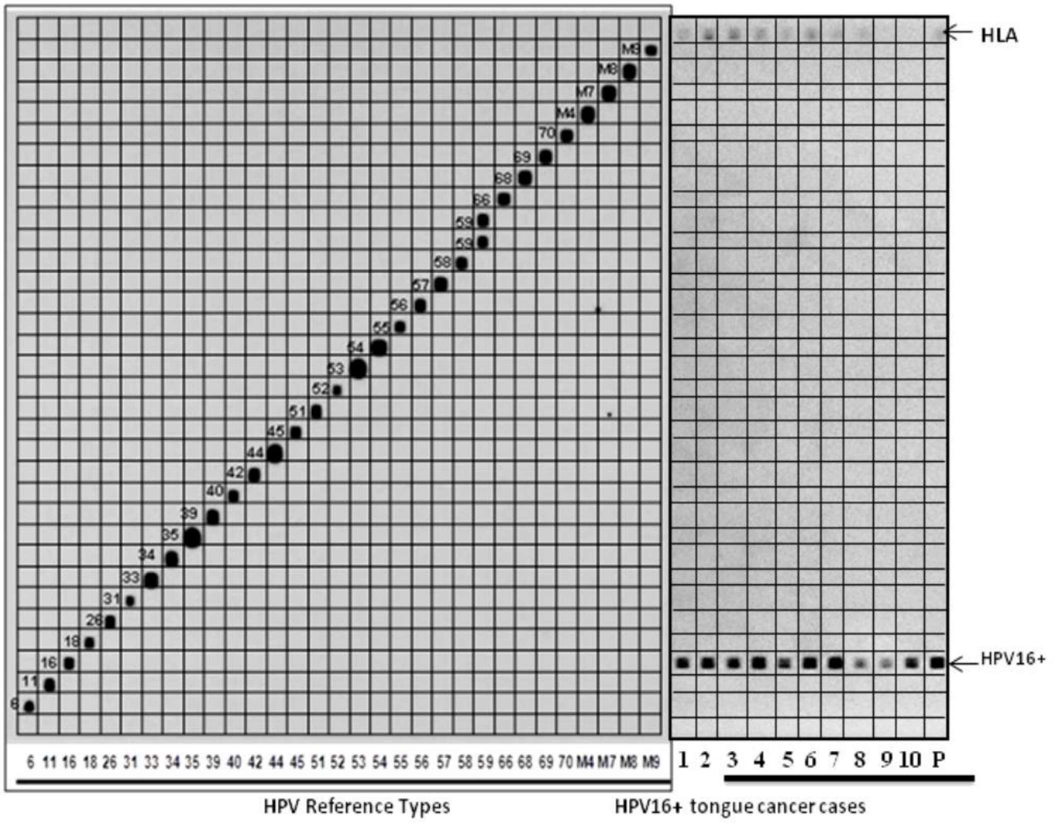

Supplementary Figure S2 (a-b): Fra-2 silencing inhibits cell proliferation. Fra-2 inhibition was accompanied by reduction in cell viability (30%) at 80nM in both (a) HPV^{+ve} (UPCI:SCC090) and (b) HPV^{-ve} (AW13516) TSCC cells but no effect in lower concentrations. Semiconfluence cancer cells transfected with indicated concentrations of Fra-2 siRNA and scrambled control for 48hrs and were harvested by trypsinization and counted for live cells by trypan blue exclusion assay. Panels a & b indicate photomicrographs of respective controls and treated cells in culture. Data represent the means ± S.D. of triplicate cultures.

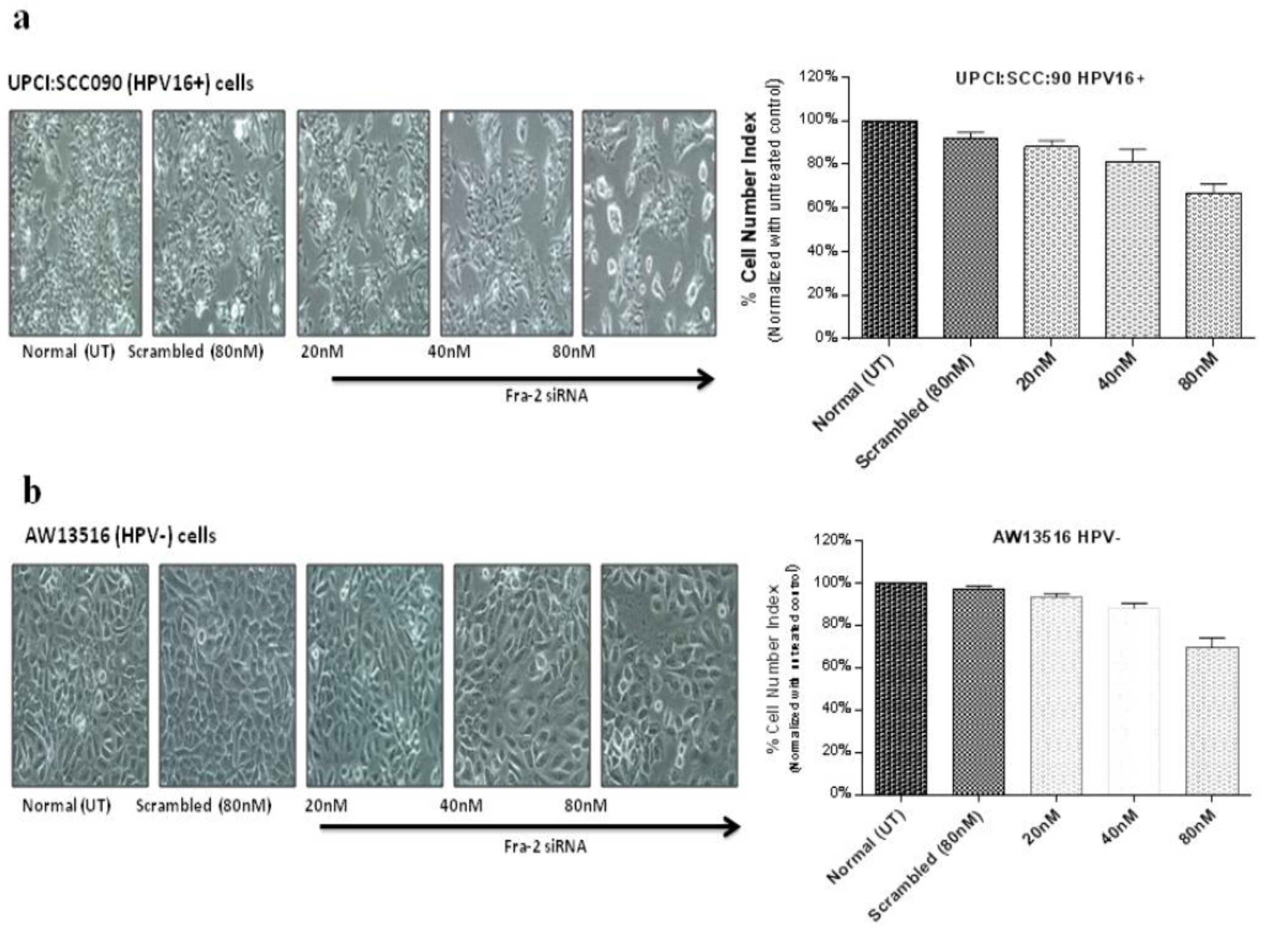

Supplementary Tables

Table 1 & 2: Primer sequences used for detection and typing of HPV sequences and mRNA transcripts analysis of AP-1 family genes

1.

Primers	Primer Sequence	References
**MY09/ MY11**	FP- 5' CGTCCMARRGGAWACTGATC-3'<br>RP- 5' GCMCAGGGWCATAAYAATGC-3'<br>Where M=A or C, W=A or T, Y=C or T and R=A or G	[1]
**HPV16 (URR)**	FP- 5' AAGGCCAACTAAATGTCAC 3'<br>RP- 5' CTGCTTTTATACTAACCGG 3'	
**β-globin**	FP- 5' GAAGAGCCAAGGACAGGTAC-3'<br>RP- 5' CAACTTCATCCACGTTACACC-3'	[2]

2.

RT-Primers	Primer Sequence	References
**c-Jun (RT)**	FP- 5' GGAGTGTCCAGAGAGCCTTG-3'<br>RP- 5' GAAAGGCTTGCAAAAGTTCG-3'	[3]
**c-Fos (RT)**	FP- 5' TTTATAGTGGGCGGAAGTGG-3'<br>RP- 5'ACGTCCTGGACAAAGGTCAC-3'	
**Fra-2 (RT)**	FP- 5' GGAGCTGGAGGAGGAGAAGT-3'<br>FP- 5' GGGCTCCTGTTTCACCACTA-3'	
**GAPDH (RT)**	FP- 5' TGGATATTGTTGCCATCAATGACC-3'<br>RP- 5' GATGGCATGGACTGTGGTCATG-3'	[4]

References

1. Prusty, B.K. & Das, B.C. Constitutive activation of transcription factor AP-1 in cervical cancer and suppression of human papillomavirus (HPV) transcription and AP-1 activity in HeLa cells by curcumin. *Int J Cancer* **113**, 951-960 (2005).
2. Das, B.C., Sharma, J.K., Gopalakrishna, V. & Luthra, U.K. Analysis by polymerase chain reaction of the physical state of human papillomavirus type 16 DNA in cervical preneoplastic and neoplastic lesions. *J Gen Virol* **73 (Pt 9)**, 2327-2336 (1992).
3. Macleod, K. *et al.* Altered ErbB receptor signaling and gene expression in cisplatin-resistant ovarian cancer. *Cancer Res* **65**, 6789-6800 (2005).
4. Shukla, S. *et al.* Aberrant expression and constitutive activation of STAT3 in cervical carcinogenesis: implications in high-risk human papillomavirus infection. *Mol Cancer* **9**, 282 (2010).
